# The Different Immunoregulatory Functions on Dendritic Cells between Mesenchymal Stem Cells Derived from Bone Marrow of Patients with Low-Risk or High-Risk Myelodysplastic Syndromes

**DOI:** 10.1371/journal.pone.0057470

**Published:** 2013-03-04

**Authors:** Zhenling Wang, Xiaoqiong Tang, Wen Xu, Zeng Cao, Li Sun, Weiming Li, Qiubai Li, Ping Zou, Zhigang Zhao

**Affiliations:** 1 Department of Hematology and Oncology, The Oncology Hospital of Tianjin Medical University, Tianjin, People’s Republic of China; 2 Department of Hematology, Institute of Hematology, Chongqing Medical University, Chongqing, People’s Republic of China; 3 Institute of Hematology and Blood Diseases Hospital, State Key Lab of Experimental Method of Hematology, Chinese Academy of Medical Sciences and Peking Union of Medical College, Tianjin, People’s Republic of China; 4 Department of Hematology, Institute of Hematology, Tongji Medical College of Huazhong University of Science and Technology, Wuhan, People’s Republic of China; University of Medicine and Dentistry of New Jersey, United States of America

## Abstract

Myelodysplastic syndrome (MDS) is a group of progressive,clonal, neoplastic bone marrow disorders characterized by hematopoietic stem cell dysregulation and abnormalities in the immune system. Mesenchymal stem cells (MSC) appear to modulate the immune system at the very first step of the immune response through the inhibition of dendritic cells (DCs) differentiation and maturation. However, it is still unclear whether the effects of MSC on the development of DCs will be altered with disease state. In addition, it is not clear whether there are differences in the effects between low-risk and high-risk MDS-MSC on DCs development. In this study, our data confirm that MDS-MSC mediate a potent inhibition of DCs differentiation. Additionaly, MDS-MSC greatly alter DCs functions, including endocytosis, IL-12 secretion, their ability to inhibit T cell proliferation. Moreover, our results show that there are major differences in DCs development and function between low-risk and high-risk MDS-MSC. Compared to high-risk MDS-MSC, low-risk MDS-MSC is characterized by a poor ability to inhibit DCs differentiation and maturation; and correspondingly, less dysfunctional DC endocytosis, mildly decreased IL-12 secretion, and a reduction in DC-mediated inhibition of T cell proliferation. Finally, our results demonstrate that MDS-MSC derived TGF-β1 is largely responsible for the inhitory effects. These results elucidate the different immunoregulatory role of MSC in low-risk and high-risk MDS on DCs development, which may be important for understanding the pathogenesis of MDS and the development of novel immune therapies for the treatment of MDS.

## Introduction

The myelodysplastic syndromes (MDS) comprise a heterogeneous group of hematological disorders characterized by peripheral blood cytopenias, due to ineffective hematopoiesis, with a high risk of transformation into acute myeloid leukemia (AML) [1]. Using the International Prognostic Scoring System (IPSS), MDS is classified into low-risk (IPSS score≤1.0) and high-risk (IPSS score>1.0) for progression towards (AML). Athough the pathogenesis of MDS remains poorly defined, accumulating evidence indicates a significant deregulation of the immune system in the complex pathogenesis of MDS [2]–[5]. This deregulation may even promote the progression of early MDS to advanced MDS [6]. Low-risk MDS is characterized by increased apoptosis in the bone marrow with autoimmune characteristics whereas high-risk MDS is associated with an immunosuppressive bone marrow microenvironment favoring for dysplastic clones and leading to disease progression [7]–[9]. In addition, there are major differences in the immune abnormalities between low-risk and high-risk MDS. Compared to low-risk MDS, high-risk MDS is associated with the presence of dysfunctional NK cells, increased Tregs, increased cytotoxic CD8+ T cells, lower apoptosis and a poor response to immunosuppressive therapy [10].

Dendritic cells (DCs), the most potent antigen-presenting cells(APCs), are key mediators for the initiation and regulation of both innate and adaptive immune responses. The ability of DCs to initiate an immune response depends on their transition from antigen-processing to antigen-presenting cells, during which they up-regulate class II major histocompatibility complex (MHCII) and T-cell costimulatory molecules (CD80, CD86) on the cell surface, a process referred to as DCs maturation [11]–[12]. This transition constitutes an important checkpoint in mounting an immune response because immature DCs not only fail to prime T cells effectively, but also serve to promote tolerance induction.

Previous studies have shown that mesenchymal stem cells (MSC) are inherently low of immunogenicity and are capable of inhibiting T cells proliferation in vitro and mediate a systemic immunosuppressive property in vivo [13]. In addition, Zhang et al and Jiang et al suggested that MSC might modulate the immune system at the very first step of the immune response through the inhibition of DCs differentiation and maturation [14]–[15]. Moreover, our previous studies demonstrated that although MDS bone marrow derived MSC (MDS-MSC) were similar to normal adult bone marrow derived MSC in morphology, growth property, surface epitopes, and differentiation ability in vitro, the immunoregulatory functions of MDS-MSC are impaired, suggesting the involvement of MSC in the pathogenesis of MDS [16]. However, it is still unclear whether the effects of MSC on the development of DCs will be altered with disease progression. In addition, the different immune abnormalities between low-risk and high-risk MDS prompt us to investigate whether there are differences in the effects of MSC on the development of DCs between low-risk and high-risk MDS. Moreover, it is unclear what the definitive role of MSC in the pathogenesis of MDS is, with respect to different phase. To this end, we investigated the effects of low-risk and high-risk MDS-MSC on the development of monocyte-derived DCs.

## Materials and Methods

### Ethics

Informed written approval was obtained from the Tianjin Cancer Hospital Institutional Review Board for these studies. All patients and healthy donors gave written consent for the clinical procedure and for the use of tissue for research purposes according to the Declaration of Helsinki.

### Isolation and Culture of MSC Derived from MDS Patients

Sixteen patients with low-risk MDS (aged from 41 to 65; IPSS score≤1.0) and fifteen patients with high-risk MDS (aged from 39 to 62; IPSS score>1.0) were investigated in this study; eight healthy donors (aged from 37 to 61) were also recruited. All patients were recently diagnosed at the time of entry into this study, and untreated at the time of study. Diagnosis was established by BM aspirate smears, BM biopsy, cytogenetic analyses and peripheral blood count criteria, according to the WHO group. After receiving informed consent from the patients according to the academic guidelines on the use of human subjects in research, human bone marrow was obtained from patients and healthy donors under the protocol approved by the institutional review board. Mononuclear cells (MNCs) were separated by a Ficoll-Paque gradient centrifugation (specific gravity 1.077 g/ml; Sigma Diagnostics, St Louis, MO, USA), and cultured in expansion medium at 37°C with 5% CO_2_ in fully humidified atmosphere. Expansion medium contained 60% DMEM/F-12 (Gibco Life Technologies, Paisley, UK), 40% MCDB-201 (Sigma), 2% fetal calf serum (FCS; Gibco), 1× insulin transferrin selenium, 1×linoleic acid bovine serum albumin, 10^−9^ M dexamethasone (sigma), 5 ng/ml basic fibroblast growth factor (Gibco), 10 ng/mL platelet-derived growth factor BB (PDGF-BB; Sigma), 10 ng/mL bone morphogenetic protein-4 (BMP-4; Sigma), 10 ng/mL insulinlike growth factor (IGF; Sigma), 100 U/mL penicillin, 1000 U/mL streptomycin (Gibco). After culture for 24–48 h, the culture medium was replaced and non adherent cells were removed. Once cells were more than 80% confluent, they were detached with 0.25% trypsin-EDTA (Sigma), then CD14 positive cells were depleted using CD14 micromagnetic beads (Miltenyi Biotec, Auburn, USA).and CD14 negative cells were replated. To ensure single cell originality of each cell colony, sorted cells were plated at concentrations of 1 cells/well in a 96 well plate coated by fibronectin (Sigma) in each setting and cultured in expansion medium. Wells with a single adherent cell were identified during the first 24 hours. The appearance of cell colonies was checked daily. Single colony was harvested by trypsinization and expanded [17]. The cloned MDS-MSC expressed CD105, CD29, CD44; they did not express hematopoietic markers CD34, CD45, or endothelial markers CD31, vWF. Moreover, CD14 and HLA-DR were also negative. They had the ability to differentiate into, at least, adipocyte, osteoblast, endothelial and neural cells in vitro [16].

### Differentiation and Maturation of DCs

Human peripheral blood mononuclear cells (hPBMCs) from healthy donors were isolated by centrifugation over Ficoll-Hypaque gradients (Nycomed Amersham, Uppsala, Sweden). Monocytes were isolated as the adherent fraction after incubation for 2 hour in RPMI 1640 (BioWhittaker, Verviers, Belgium) supplemented with 10% fetal calf serum (FCS) (BioWhittaker), 100 U/mL penicillin/streptomycin (Bristol-Myers Squibb, Sermoneta, Italy), and 50 µM 2 mercaptoethanol (Bio-Rad, Segrate, Italy) (DC medium) at 37°C. Then, CD14+ monocytes were obtained by using the CD14 micromagnetic beads (Miltenyi Biotec, Auburn, USA). After extensive washing, adherent monocytes were differentiated into DCs by culture in 10 ng/mL recombinant human IL-4 (rhIL-4) (R&D Systems, Minneapolis, MN) and 100 ng/mL recombinant human GM-CSF (rhGM-CSF) (Schering-Plough, Kenilworth, NJ) in DC medium. To induce maturation of monocyte-derived cells, lipopolysaccharide (LPS) (1 µg/mL) was added for another 48 hours of culture with GM-CSF and IL-4.

### Preparation of T cell Subsets

hPBMCs from healthy donors were isolated by centrifugation over Ficoll-Hypaque gradients (Nycomed Amersham, Uppsala, Sweden). CD4+ T lymphocytes were isolated from hPBMCs by using CD4 micromagnetic beads (Miltenyi Biotec, Auburn, USA) according to the manufacturer's instructions. CD4+ cell purity was 97% ±2%.

### Coculture Experiment

Irradiated MSC (15 Gy) were added to 6-well plates at a density of 1×10^5^ cells per well unless otherwise indicated. To prevent the contamination of MSC with harvested monocyte-derived cells, transwell chambers with a 0.4 um pore size membrane (Corning) were used. MSC were cultured on the reverse side of the membrane in the chamber. After obtaining a confluent feeder layer, monocytes were seeded on the upper side in the membrane of the chamber. To study the influence of MSC on DCs differentiation, monocytes were cultured for 7 days in the presence or absence of MSC. To study the effects of MSC on DCs maturation, immature DCs were co-cultured with MSC for 48 h before maturation. In some experiments, 1-methyltryptophan, indomethacin, 1, 4 PBIT, anti-rhIL-6 mAbs, anti-rhTGF-β1 mAbs were added at the beginning of the culture.

### FACS Analysis

For immunophenotype analysis, cultured cells were washed with PBS containing 0.5% bovine serum albumin (BSA, Sigma), and incubated with primary antibodies (10–20 ng/ml) for 30 minutes at 4°C. Primary antibodies included mAb against CD1a, CD4, CD14, CD40, CD80, CD83 and CD86 (BD Biosciences Pharmingen, San Diego, CA, USA). We used same-species, same-isotype irrelevant antibody as negative control. Cell analysis was performed with FACS Calibur system using Cellquest software. For phenotypic analysis of MSC, the anti-CD29, anti-CD31, anti-CD34, anti-CD44, anti-CD45, anti-CD105, anti-vWF and anti-HLA-DR mAbs were purchased from BD Biosciences Pharmingen (San Diego, CA, USA). MSC phenotype was analyzed by indirect single fluorescence analysis.

### Cytokine Analysis

DCs were washed and plated in a volume of 1 ml of culture medium 24-well plates in the presence or absence of irradiated MSC. After 24 h, IFN-γ(200 U/ml) and LPS (100 ng/ml) were added. Cell-free supernatants were harvested 48 h later. Levels of IL-12 were measured using ELISA kits (R&D Systems).

### Endocytosis Assay

Endocytosis was measured as the cellular uptake of fluorescein isothiocyanate (FITC)–dextran and was quantified by flow cytometry. Approximately 5×10^5^ cells per sample were incubated in medium containing FITC-dextran (1 mg/mL; molecular weight 40000; Sigma, St Louis, MO) for 60 minutes. After incubation, cells were washed twice with cold phosphate buffered saline (PBS) to stop endocytosis and remove excess dextran. Cell were then fixed in cold 1% formalin. The quantitative uptake of FITC-dextran by the cells was determined by FACS. At least 10000 cells per sample were analyzed.

### Mixed Leukocyte Reaction

Allogeneic CD4+ T cells were purified from hPBMC by using the MACS CD4 isolation kit. In order to test the HLA matching between suppressor and responder, all HLA-A, -B, -C and -DR alleles typing has been performed by sequencespecific oligonucleotide probe (SSOP) methods. Always mismatches at HLA-A, -B, -C, and -DR were found between CD4+ T cells and DCs. CD4+T cells resuspended at 1×10^6^ cells/well were added to wells with or without irradiated (15 Gy) allogeneic suppressive cells. The culture was continued and 3H-thymidine was added 18 hours before the end of the 120-hour culture. The T-cell proliferation was represented as the incorporated radioactivity in cpm and shown as mean±SD of triplicate values.

### Statistical Analysis

The results were statistically analyzed using the SPSS11.0 statistical package (SPSS Inc, Chicago, IL). The Student t test for paired data (2-tail) and ANOVA was used to test the probability of significant differences between samples.

## Results

### MSC Inhibit DCs Differentiation and Maturation from Monocytes

**Figure 1:**
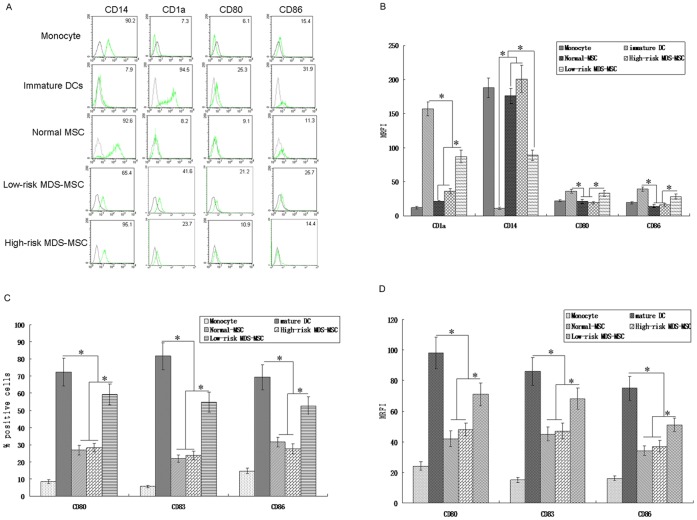
MDS-MSC inhibit the differentiation and maturation of monocyte derived DCs. (A) Monocytes **(1×10^6^)** were cultured with medium alone or with IL-4 and GM-CSF in the presence or absence of MSC **(1×10^5^)**. After 7 day's culture, the expression of CD1a, CD14, CD80 and CD86 was analyzed by FACS. (B) The data represent MRFI of five individual experiments (mean±SD). (C) Monocyte-derived immature DCs **(1×10^6^)** were further cultured in media alone (Control) or with TNF-a (200 U/ml) in the presence or absence of MSC **(1×10^5^)**. After 48 h, cells were collected, the expression of CD80, CD83 and CD86 was analyzed by FACS. Numbers in histograms indicate the mean fluorescence of each cell population. (D) The data represent MRFI of five individual experiments (mean±SD). Results are expressed as mean±SD of triplicates of 5 separate experiments.*P≤0.05.

CD14+ monocytes were cultured in the presence of IL-4 and GM-CSF with or without MSC coculture. At the end of 7 days culture, CD14+ monocytes gave rise to the typical marker of immature DCs, displaying increased expression of CD1a in contrast with decreased or absence of CD14 expression. To investigate the effect of MSC on DCs, irradiated MSC were co-cultured with freshly isolated monocytes together with GM-CSF and IL-4 (MSC:monocyte 1∶10). Our data indicated that both normal-MSC and MDS-MSC (including low-risk MDS-MSC and high-risk MDS-MSC) could inhibit monocyte differentiation into immature DCs. Given that high-risk MDS-MSC and low-risk MDS-MSC might exact differences on the functions of DCs differentiation, we examined these functions of high-risk MDS-MSC and low-risk MDS-MSC. Compared with high-risk MDS-MSC, low-risk MDS-MSC had little effect on CD1a, CD14, CD80 and CD86 expression (Figure 1A–B), indicating that the suppressive function of low-risk MDS-MSC on DCs differentiation was less than that of high-risk MDS-MSC. Furthermore, our results showed that MSC could inhibit the maturation of DCs. As shown in Figure1C, the expression of the costimulatory molecules CD80 and CD86 and of the maturation marker CD83 were significantly lower in cells cultured in the presence of MSC. Compared with high-risk MDS-MSC, low-risk MDS-MSC had little effect on CD80, CD83 and CD86 expression, indicating that the effect of low-risk MDS-MSC on DCs maturation was weaker than that of high-risk MDS-MSC (Figure 1C–D). At last, our results indicated that the effect of MDS-MSC on DCs maturation was similar to that of normal-MSC.

### MSC Inhibit the Endocytosis of Monocyte Derived DCs

**Figure 2:**
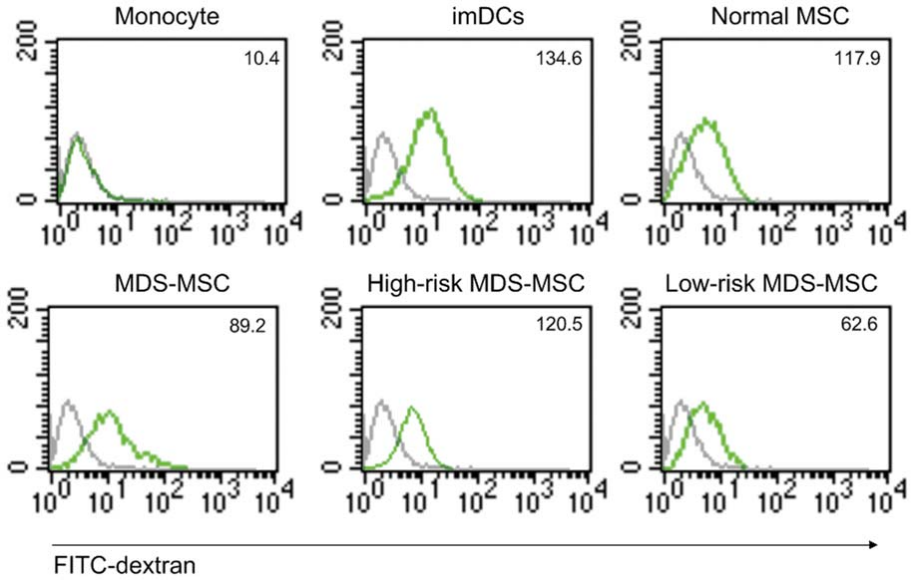
MDS-MSC inhibit the endocytosis of monocyte derived DCs. DCs **(5×10^5^)** were incubated in medium with FITC-labeled dextran at a concentration of 1 mg/ml in the presence of MSC **(5×10^4^)** at day 7. After an incubation period of 60 min at 37°C (green line) or 4°C as a control (gray line), cells were harvested and analyzed by FACS.Numbers in histograms indicate the mean fluorescence of each cell population. Results are expressed as mean±SD of triplicates of 5 separate experiments. *P≤0.05.

To investigate the effect of MSC on DCs endocytosis, immature DCs were exposed to MSC 48 h before DCs were incubated with fluorescein isothiocyanate (FITC)-labeled dextran. Compared with the DCs alone group, endocytic activity was significantly reduced in co-culture with MSC. In addition, we found that the effect of MDS-MSC on DCs endocytosis was weaker than that of normal-MSC (Figure 2). However, the effect of high-risk MDS-MSC on DCs endocytosis was similar to that of normal-MSC, and the effect of low-risk MDS-MSC was even weaker (Figure 2), implying that the reduced effect of MDS-MSC on DCs endocytosis was due to the low-risk MDS-MSC portion.

### MSC Suppressed IL-12 Production by DCs

**Figure 3:**
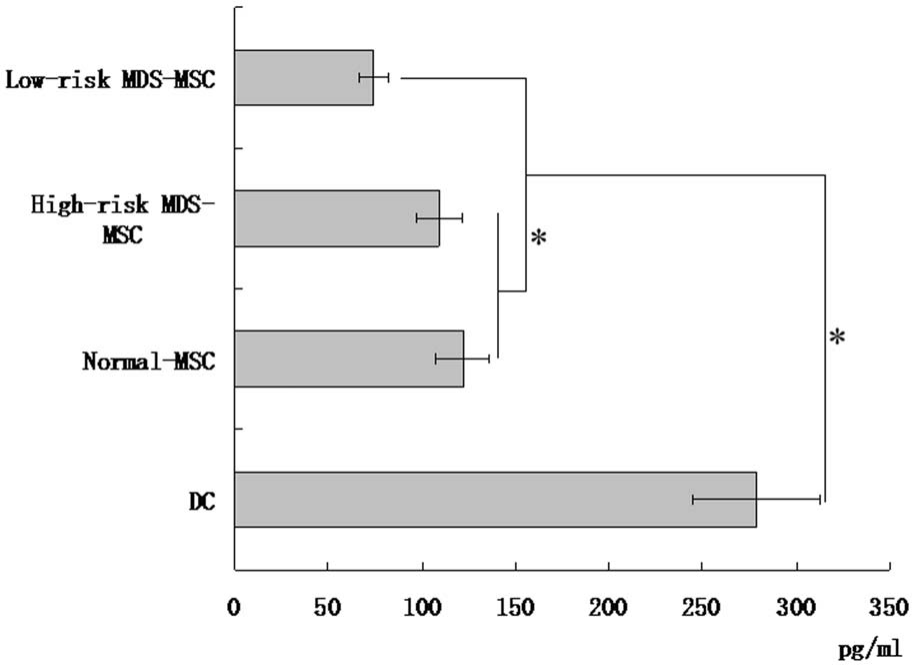
MDS-MSC reduce IL-12 secretion of monocyte-derived DCs. DCs **(1×10^6^)** obtained from monocytes after 7 days of induction with GM-CSF plus IL-4 were stimulated by LPS for an additional 48 hours with or without MSC coculture, and then cell-free supernatants were collected and quantified by ELISA for IL-12 production. Results are expressed as mean±SD of triplicates of 5 separate experiments. *P≤0.05.

Because the secretion of IL-12 is critical for the maturation and function of DCs, we next investigated whether MSC can interfere with IL-12 production by DCs. As shown in Figure 3, cells cocultured with MSC produced low amounts of IL-12 (121.8±14.4 pg/mL for normal-MSC, 108.9±12.4 pg/mL for high-risk MDS-MSC, 74.3±8.1 pg/mL for low-risk MDS-MSC, respectively), whereas much higher amounts (278.6±34.2 pg/mL) were released by cells cultured alone. The effect of low-risk MDS-MSC on the capacity of monocyte-derived DCs to secrete IL-12 was weaker than that of high-risk MDS-MSC or normal-MSC (Figure 3).

### MSC Inhibit the Ability of Monocyte-derived DCs to Induce T-lymphocyte Proliferation

**Figure 4:**
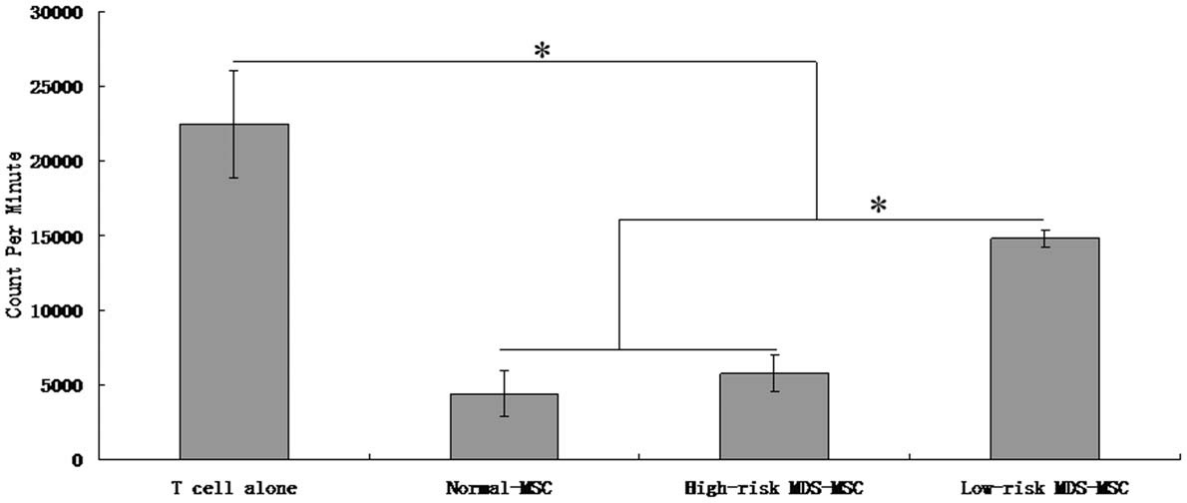
MDS-MSC inhibit the capability of inducing T-cell response in MLR of monocyte-derived DCs. mDCs **(1×10^5^)** were irradiated and used to induce allogeneic T cells (10^6^ responder cells/well) with or without MSC **(1×10^5^)** in the MLR culture. After culture for 5 days, T-cell proliferation was evaluated by adding 3H-thymidine to each well 18 h before the cultures were terminated. Results are expressed as mean±SD of triplicates of 5 separate experiments. *P≤0.05.

In this study, our results demonstrated that MSC could inhibit the proliferation of T cells induced with monocyte derived DCs. As shown in Figure 4, there was a significant reduction in T cell proliferation induced by DCs, in the presence of irradiated MSC (MSC/T-lymphocyte ratio was 1∶10). However, the immunosuppressive rate of MDS-MSC on T cell proliferation was less than that of normal-MSC (53.6±4.6% in MDS-MSC versus 80.5±6.28% in normal derived MSC, p<0.05), suggesting that the immunosuppressive effects on T cell proliferation of MDS-MSC were impaired. Next, we examined the immunoregulatory functions of high-risk MDS-MSC and low-risk MDS-MSC. We found that although both high-risk MDS-MSC and low-risk MDS-MSC could inhibit the proliferation of T cells, the immunosuppressive rate of low-risk MDS-MSC on T cell proliferation was less than that of high-risk MDS-MSC (33.8±2.8% in low-risk MDS-MSC versus 74.3±5.4% in high-risk MDS-MSC, p<0.05). Moreover, the immunosuppressive rate of high-risk MDS-MSC on DC-induced T cell proliferation was slightly lower than that of normal-MSC (74.3±5.4% in high-risk MDS-MSC versus 80.5±6.28% in normal derived MSC), but not statistically significant (p>0.05) (Figure 4).

### TGFβ1 Plays a Key Role in the MSC-mediated Inhibition of DCs Differentiation

**Figure 5:**
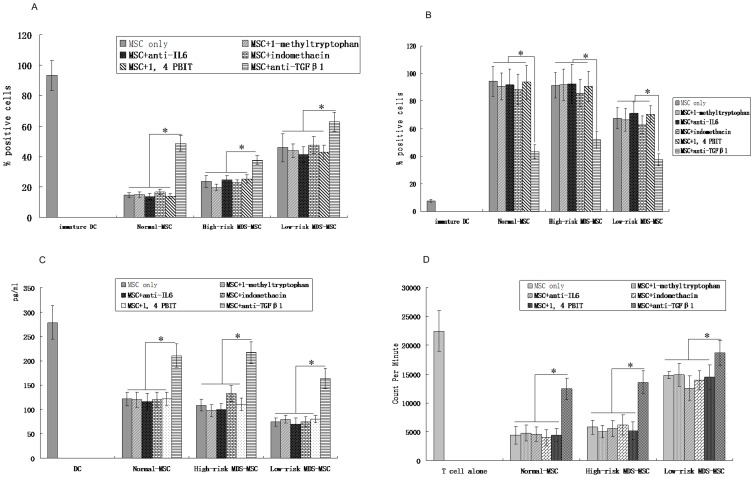
TGFβ1 plays a key role in the MSC-mediated inhibition of DCs differentiation and functions. Monocytes were cultured with GM-CSF and IL-4 to induce differentiation into DCs. Cultures were performed either in the absence or in the presence of MSC. In addition, anti-rhTGF-β1, or anti-rhIL-6, or 1-methyltryptophan, IDO inhibitor, or indomethacin, PGE2 inhibitor, or PBIT, NO inhibitor was added to monocyte-MSC cocultures. After 5 days, expression of CD1a (A) and CD14 (B) in cells cultured under the described was performed to check DCs differentiation. (C) IL-12 was measured in culture supernatants after 48-hour stimulation with LPS of monocyte-derived cells cultured for 7 days with GM-CSF and IL-4 either in the absence or in the presence of MSC., anti-rhTGF-β1, or anti-rhIL-6, or 1-methyltryptophan, IDO inhibitor, or indomethacin, PGE2 inhibitor, or PBIT, NO inhibitor was added at the beginning of the culture. DCs obtained from monocytes after 7 days of induction with GM-CSF plus IL-4 were stimulated by LPS for an additional 48 hours without MSC coculture. (D) anti-rhTGF-β1, or anti-rhIL-6, or 1-methyltryptophan, IDO inhibitor, or indomethacin, PGE2 inhibitor, or PBIT, NO inhibitor was added at the beginning of MLR coculture. T-lymphocyte proliferation was assessed by [3H]-thymidine incorporation. Data are expressed as mean±SD of triplicates of 5 separate experiments. *P≤0.05.

TGFβ1 has been shown to modulate a variety of immune functions in vitro [18]. Our previous study also showed that MDS-MSC secreted more IL-6 but less TGF-β1, compared to normal-MSC [19]. In addition, we found that MDS-MSC take part in the regulation of the immune response by secreting TGFβ1. In this study, we investigated whether TGFβ1 was related to the effect of MSC on DCs differentiation. To this aim, a neutralizing anti-TGFβ1 antibody was added on day 0, at the beginning of the culture, or on day 7, and DCs maturation was induced by addition of LPS. Our results showed that the addition of anti-TGFβ1 during the process of maturation did not change the phenotypic profile of DCs. On the contrary, when anti-TGFβ1 was added during the overall period of culture, cells displayed high CD1a and low CD14 expression compared with cells cultured with MSC in the absence of the inhibitor (Figure 5A–B). In parallel, IL-12 secretion was significantly increased by the addition of anti-TGFβ1(Figure 5C). Moreover, analysis of DCs function revealed that, in the presence of anti-TGFβ1, there was a partial restoration of the ability of cells to stimulate T-cell response in MLR (Figure 5D). At last, because previous studies reported that IL-6 or PGE2, produced by MSC, would play a major role in the MSC-mediated inhibition on DCs differentiation [20]–[21], we investigated whether IL-6 or PGE2 might have an impact on DCs differentiation and function. As shown in Figure 5, neither IL-6 nor PGE2 plays a role in the MSC-mediated inhibition on DCs maturation or function. Our data also show that IDO and NO are not involved in the MSC-mediated inhibition of DCs maturation and function. Taken together, this data strongly suggests that TGFβ1 plays a major role in the MSC-mediated inhibition of DCs differentiation and function.

## Discussion

In the present study, we analyzed the effect of low-risk and high-risk MDS-MSC on monocyte derived DCs maturation and differentiation. We confirm that MDS-MSC mediate a potent inhibition on DCs differentiation. However, there are great differences between normal-MSC or MDS-MSC on DCs functions, including the effect on DCs endocytosis, IL-12 secretion, and inhibition of T cell proliferation. More importantly, we show that there are major differences between low-risk and high-risk MDS-MSC on DC differentiation and function. Compared to high-risk MDS-MSC, low-risk MDS-MSC is associated with a poor ability to inhibit DCs differentiation and maturation, dysfunctional DCs endocytosis, decreased IL-12 secretion, and DC-mediated inhibition of T cell proliferation. Finally, our results demonstrate that MDS-MSC derived TGF-β1 is largely responsible for these inhibitory effects.

Dysregulation of the immune response has been found to be relevant for the pathogenesis of bone marrow failure conditions, such as Aplastic Anaemia and a sub-group of MDS [22]. In addition, current knowledge indicates that aberrant immune responses and T-cell-mediated inhibition of hematopoiesis have been associated with the pathophysiology of MDS [23]–[24]. One of the most intriguing and promising features of MSC is their capacity to escape immune recognition and to inhibit several functions of the immune system. Previous studies have shown that MSC are capable of inhibiting T cell proliferation in vitro and mediate a systemic immunosuppressive property in vivo [13], [15], [25]–[26]. MSC have also been shown to exert a potent suppression on both innate and adaptive immunity by acting on NK and B lymphocytes and DCs [14]–[15], [27]–[28].

Our previous study showed that MSC derived from MDS (MDS-MSC) were similar to normal adult bone marrow derived MSC in morphology, growth property, surface epitopes, and differentiation ability in vitro. In addition, MDS-MSC had normal karyotype and ultrastructure. However, MDS-MSC showed reduced hematopoiesis support function, as compared to their normal counterparts. Moreover, the capacity of MDS-MSC to inhibit T lymphocyte activation and proliferation was impaired in vitro [16]. These results indicate that MDS-MSC have impaired immunomodulatory and hematopoiesis support functions, suggesting their critical role in the pathogenesis of MDS. Lastly, MSC represent an important cellular component in the bone marrow microenvironment. All these data indicate that MSC play a prominent role in the immune abnormalities and pathogenesis of MDS.

It is well known that DCs are important immune cells and key participants in MLR and the magnitude and direction of the immune response in vivo [29]–[30]. Although previous studies demonstrated that normal adult derived MSC could inhibit differentiation and function of monocyte-derived dendritic cells [14]–[15], the definitive role of MDS-MSC on the development of DCs was still unclear. In this study, our results indicated that the effect of MDS-MSC on DCs differentiation and maturation was similar to that of normal-MSC. Our results showed that both normal-MSC and MDS-MSC treated monocytes displayed low expression of CD1a and persistently high expression of CD14, indicating that the cells had not differentiated into immature DCs phenotypically. Furthermore, our results indicated that the effect of MDS-MSC on DCs differentiation and maturation was similar to that of normal-MSC, the expression of the maturation marker CD83 was significantly lower in cells cultured in the presence of normal-MSC or MDS-MSC. However, there were major differences between normal-MSC or MDS-MSC on DCs functions. Firstly, we found that the effect of MDS-MSC on DCs endocytosis was weaker than that of normal-MSC; Secondly, our data showed that DCs cocultured with MDS-MSC produced low amounts of IL-12 (95.7±7.3 pg/mL), whereas much higher amounts of IL-12 were released by DCs cultured with normal-MSC (121.8±14.4 pg/mL); Lastly, the immunosuppressive ability of MDS-MSC on T cell proliferation was less than that of normal-MSC (53.6±4.6% in MDS-MSC versus 80.5±6.28% in normal derived MSC, p<0.05), suggesting that the immunosuppressive effects on T cell proliferation of MDS-MSC were impaired. All these results imply that there are great differences between normal-MSC and MDS-MSC on DCs functions.

Previous studies have demonstrated that low-risk MDS and high-risk MDS possess different biological characteristics [11]. Our previous data showed that there are major differences in the immune abnormalities between low-risk and high-risk MDS-MSC. Compared to low-risk MDS-MSC, high-risk MDS-MSC is associated with the presence of increased Tregs, higher apoptosis, higher immunosuppressive rate, and a poor ability of hematopoietic support (in press). In this study, we found that there were several differences between low-risk and high-risk MDS-MSC on DCs differentiation, maturation and function. Firstly, our data showed that the suppressive function of low-risk MDS-MSC on DCs differentiation was less than that of high-risk MDS-MSC; Secondly, compared with high-risk MDS-MSC, low-risk MDS-MSC had little effect on CD80, CD83 and CD86 expression, indicating that the effect of low-risk MDS-MSC on DCs maturation was weaker than that of high-risk MDS-MSC; Thirdly, the function of low-risk MDS-MSC on DCs endocytosis was weaker than that of high-risk MDS-MSC; Fourthly, IL-12 plays a key role in the maturation and function of DCs. In additon, insufficient IL-12 production of DCs has been implicated in the induction of anergy and tolerance of T cells. In this study, the function of low-risk MDS-MSC on the capacity of monocyte-derived DCs to secrete IL-12 was weaker than that of high-risk MDS-MSC (108.9±12.4 pg/mL for high-risk MDS-MSC, 74.3±8.1 pg/mL for low-risk MDS-MSC, respectively, p<0.05); Finally, the immunosuppressive ability of low-risk MDS-MSC on T cell proliferation was less than that of high-risk MDS-MSC (33.8±1.8% in low-risk MDS-MSC versus 74.3±5.4% in high-risk MDS-MSC, p<0.05). All the differences showed that the inhibitory effect of low-risk MDS-MSC on DCs functions was weaker than that of high-risk MDS-MSC, suggesting a significant deregulation of the immune response in the complex pathogenesis of MDS.

The mechanism by which MSC exerts their inhibitory effect on DCs function is still poorly defined. There are several mechanisms that may account for their regulatory properties. Jiang et al suggested that MSC-derived IL-6 and macrophage colony stimulating factor (M-CSF) could be responsible for the observed inhibitory effect [15]. Djouad et al also suggested that IL-6 was involved in the immunoregulatory mechanism mediated by MSC through a partial inhibition of DCs differentiation [21]. In addition, others demonstrated that PGE2 played a major role in MSC-mediated inhibition of DCs function [20]. Although high levels of IL-6 were secreted by MDS-MSC in our experiments, this did not affect the inhibitory function of MDS-MSC on DCs. Also, our data indicated that PGE2 played no role in the MSC-mediated inhibition of DCs maturation and function. Our results also suggest that TGF-β1, produced by MSC, plays a major role in the MSC-mediated inhibition of DCs differentiation, instead of NO or IDO. The discrepancies between the reports might be attributed to the distinct functional features of MDS-MSC, different experimental protocols, or even dissimilar cell populations. TGF-β1 is a protein that controls proliferation, cellular differentiation, and other functions in most cells [31]. It also plays a role in immunity and cancer [32]–[34]. Our previous studies demonstrated that MDS-MSC could inhibit T cell proliferation by secreting TGF-β1 [35]. These results clearly showed that MDS-MSC displayed their inhibitory function in a novel way: secreting TGF-β1.

In summary, we investigate for the first time the immunoregulatory functions of MSC on DCs in both low- and high-risk MDS at the single cell level. Our data suggest that MDS-MSC may modulate the immune system, not only acting directly on T cells but also at the first step of the immune response through the inhibition of DCs differentiation and maturation. More valuably, we find that there are great differences between low-risk and high-risk MDS-MSC on DCs differentiation and maturation. As a corollary, the different effects between low-risk and high-risk MDS-MSC not only help us to further elucidate the etiology and pathology of MDS, but also help us to find new therapeutic strategies for the treatment of MDS.
